# A Case of Severe Bullous Dermatitis With Mixed Bullous Pemphigoid and Pemphigus Vulgaris Cutaneous Manifestations

**DOI:** 10.7759/cureus.20433

**Published:** 2021-12-15

**Authors:** Reece D Burns, Develyn Vetos, Richard Muraga

**Affiliations:** 1 Internal Medicine/Pediatrics, University of Kansas Medical Center, Wichita, USA; 2 Internal Medicine/Dermatology, University of Kansas Medical Center, Wichita, USA; 3 Internal Medicine, University of Kansas School of Medicine, Wichita, USA

**Keywords:** bullous skin disease, bullous dermatoses, bullous lesion, autoimmune bullous dermatoses, autoimmune bullous disorders, atypical non bullous pemphigoid, bullous

## Abstract

Bullous dermatoses include the rare, chronic autoimmune diseases pemphigus vulgaris and bullous pemphigoid. These diseases are traditionally taught to be differentiated by the presence of mucosal lesions (pemphigus vulgaris) and bullae without mucosal involvement (bullous pemphigoid). In the clinical setting, however, these diseases often contain overlapping features that present challenges to care teams without access to dermatologic care and leave patients without a clear treatment pathway. The ability to differentiate these two diseases clinically is imperative as it determines treatment regimens which when applied can mitigate unnecessary morbidity and mortality. Identifying these conditions clinically for the correct treatment also allows providers to rely less on laboratory assessments which are often unavailable or may take considerable time to result. This report details the clinical course of a patient who presented with an undifferentiated bullous dermatitis with features of both pemphigus vulgaris and bullous pemphigoid and aims to highlight the features of presentation which overlap between pemphigus vulgaris and bullous pemphigoid and those which are more characteristic for one over the other.

## Introduction

Bullous dermatoses include the chronic autoimmune diseases pemphigus vulgaris and bullous pemphigoid. Pemphigus vulgaris (PV) pathology arises when autoantibodies to keratinocyte proteins desmoglein 1 and desmoglein 3 are produced and cause destruction of the keratinocyte structure [1]. The resultant clinical manifestations result in skin and mucosal vesicles that unroof easily under pressure and become ulcerative [1]. Oral lesions occur in upto 90% of PV cases throughout the disease course [1]. PV is a rare disease that typically presents in middle-aged individuals, with higher incidence rates in Ashkenazi Jewish and Middle Eastern populations [1].

Bullous pemphigoid (BP) is the most common autoimmune blistering disorder [2]. It affects adults most commonly after the eighth decade and rarely affects children or adolescents [2]. European populations tend to have the highest incidence rates [2]. BP arises as a result of autoantibody formation to basement membrane antigens (BP180 and BP230) that comprise the hemidesmosomes which connect the epidermis and dermal layers of the skin [2]. Disease activity correlates with the quantity of circulating levels of antibodies to these components. It presents with localized or widespread pruritic, tense blisters most commonly on the abdomen, inner thighs, inguinal areas, and axilla. The lesions may rest on erythematous or edematous backgrounds [2]. While not classically associated with or taught, 10%-30% of BP cases present with mucosal lesions [2]. 

This presentation details the clinical course of a patient who presented with an undifferentiated bullous dermatitis with features of both pemphigus vulgaris and bullous pemphigoid. Prior workup and skin biopsies had been equivocal. As PV and BP have differing treatment options, prompt diagnosis is essential to improve quality of life and decrease morbidity and mortality. The aim of this report is to detail a complex clinical scenario in which definitive diagnostics and specialty care are not available and treatment must be initiated in timely fashion based on clinical deduction to avoid unnecessary morbidity and mortality. 

## Case presentation

**Figure 1:**
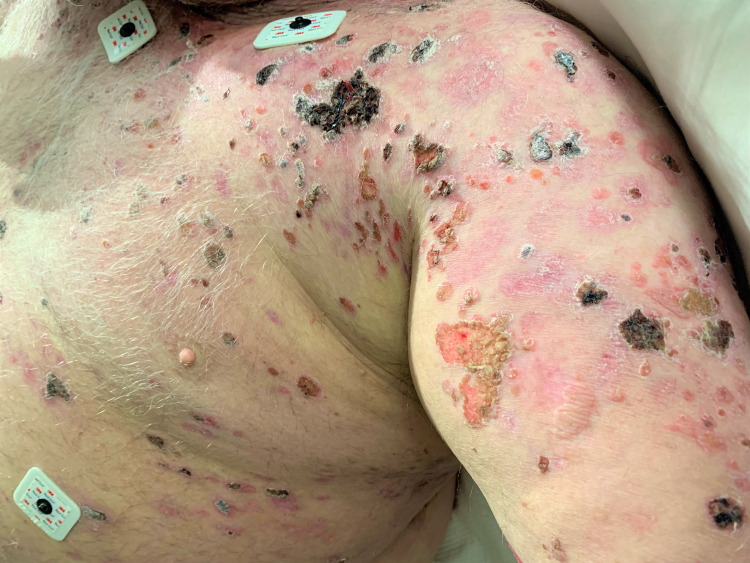
Mixed new and healing unroofed lesions over the left torso and arm.

**Figure 2:**
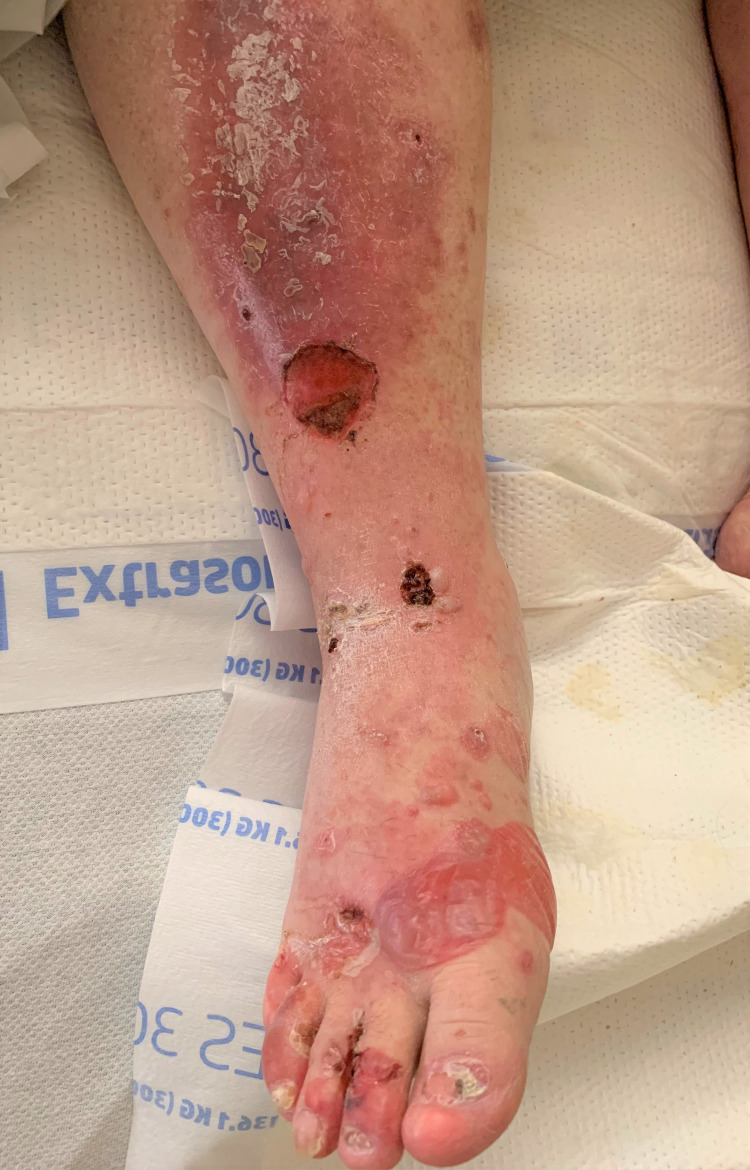
Prominent pedal bullae and unroofed lesions.

**Figure 3:**
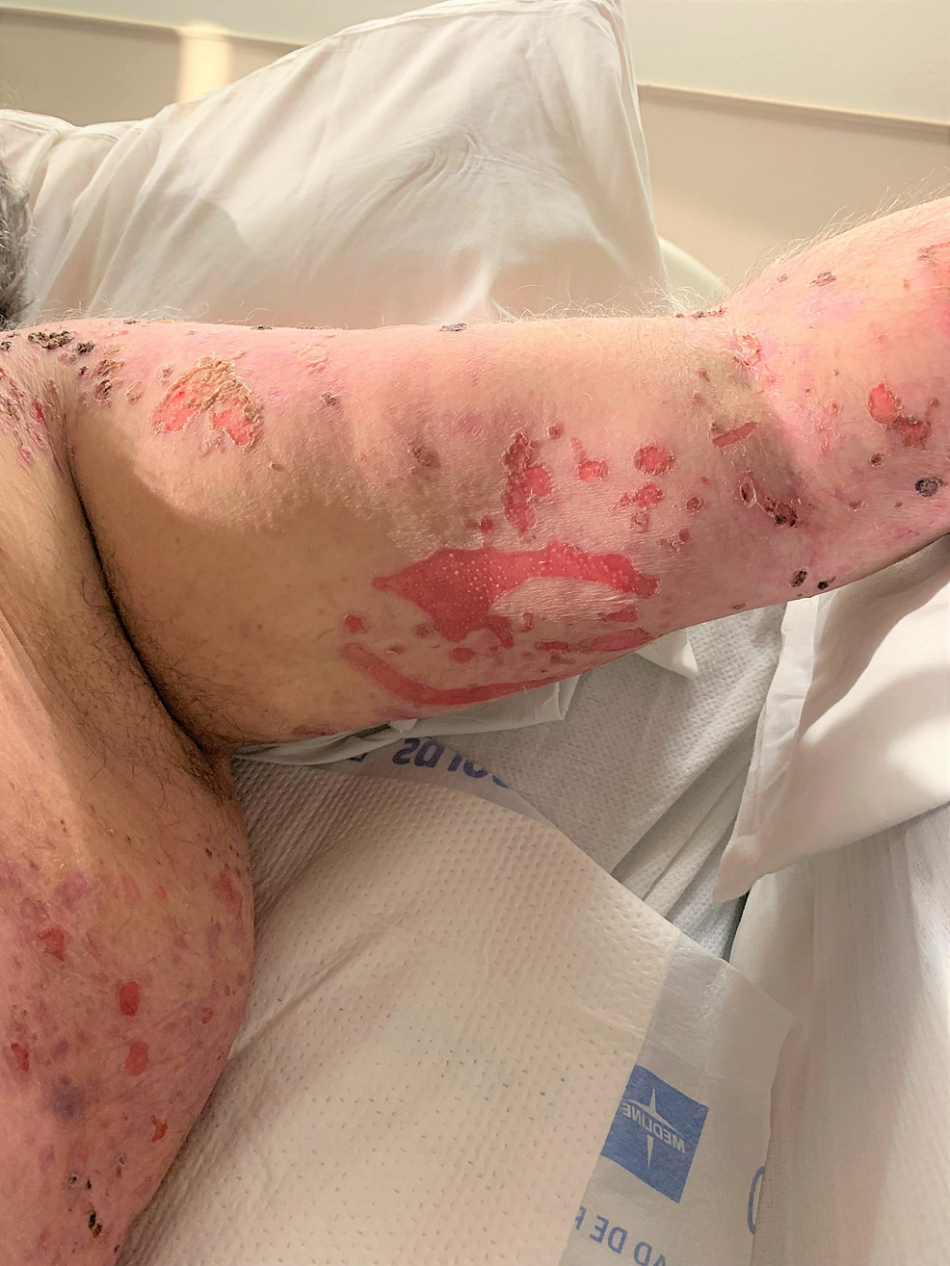
Exposed mucosal lesion area causing significant discomfort with movement.

A 63-year-old male was admitted to the inpatient unit due to intractable pain and undifferentiated skin lesions refractory to treatment for the past three years. Disseminated lesions at various stages include several large tense bullous lesions that were not easily broken in scattered areas (particularly the feet) and numerous painful and unroofed ulcerative lesions which covered more than 90% of his body surface area and oral mucosa (Figures 1, 2). Unroofed bullae drained sanguineous and purulent fluid and caused significant discomfort upon movement. The patient’s largest area of complaint was the axillary folds and inner arms, both of which were covered in ulcerated bullae (Figure 3). The patient was in significant mental distress due to his increasingly poor quality of life as his condition progressed.

The patient’s clinical course was complicated by an inconsistent and unclear coordination of care and diagnosis. His three-year history of undifferentiated bullous pemphigoid versus pemphigus vulgaris was treated with 200 mg of hydroxychloroquine daily along with reported previous methotrexate, mycophenolate mofetil use, and a daily 2.5 mg dose of prednisone. The cutaneous and oral lesions had not been well controlled for the past year and had significantly worsened in the past three weeks. Previous point-of-care skin punch biopsies were reportedly unable to identify the underlying pathology. Emergency department punch biopsy records reported an undifferentiated blister with extensive granulation tissue.

Three weeks prior to presentation, the patient’s prednisone therapy was increased to 20 mg daily without improvement. Upon admission, he was started on empiric antibiotic therapy due to the extent of exposed mucosa but was deescalated to observation after active infection was ruled out. The patient was started on 60 mg IV methylprednisolone twice daily. Hydroxychloroquine was discontinued per rheumatology’s recommendations due to its predisposition to contribute to flares. Recommendations from the wound care team and dermatology included cleansing unroofed bullas with normal saline and dressing with low-adherence contact layers. Moisture-wicking fabrics and adaptable patient bedding with control over pressure points were also recommended. 

Serologic workup for BP antibodies BP180 and BP230, as well as PV-associated antibodies to desmoglein 1 and desmoglein 3, was performed during the patients hospital admission and sent to a specialty laboratory. Weeks later, antibodies to BP180 were reported significantly elevated, while BP230, desmoglein 1, and desmoglein 3 were within normal limits. The patient followed up with dermatology practice and was placed on high-dose oral corticosteroids with plans to escalate to rituximab if adequate disease control was not attained. At the time of follow-up after several weeks of systemic steroid treatment, the patient had no new lesions and the flare was considered stable. The patient was further lost to follow-up.

## Discussion

The classically taught presentation of PV vs BP is usually distinguished by the presence of tense bullae (BP) or mucosal lesions (PV). In this case, the patient presented with a mixed clinical presentation, which made it difficult to distinguish pathology and initiate a care plan. While laboratory evaluation is definitive, it is often unable to be attained in timely fashion, particularly in an urgent clinical presentation as in this case. This puts more emphasis on physical exam findings by a multidisciplined care team. In patients such as this, dermatologic referral is always warranted. However, specialty referral is often not readily available (as in this case). In instances of acute flares with minimal information or unclear medical history, the presence of persistently tense bullae on exam can be a key finding to strongly guide diagnosis to BP over PV, even if bullae are in the minority of findings. Due to PV pathogenesis causing ulcerating, desquamating blisters, there is no structural support in the skin for persistently tense bullae [2]. Definitive diagnosis is always recommended to be made with a combination of serology and immunofluorescence staining [3-4].

This quick clinical key differentiation of BP and PV is crucial as therapeutic management varies, sometimes widely. Bullous pemphigoid treatment focuses on controlling pruritus and decreasing formation of blisters [5]. Initial therapies consist of topical corticosteroids, systemic steroids, or doxycycline. High potency topical steroids are highly effective in treatment but may pose difficulties for the patient if needing widespread application, particularly if there is no caregiver to assist [5-8]. For this reason, oral corticosteroids or doxycycline is often chosen, both of which have their own benefits and risks. Doxycycline has fewer long-term side effects but is slower to induce remission of flares [5-8]. Severe disease is often treated first with oral steroids [5-8]. Methotrexate has limited evidence for effectiveness as a first-line therapy. Severe or widespread lesions may warrant the addition of a corticosteroid-sparing agent such as methotrexate, mycophenolate, or azathioprine [5-8]. Refractory cases see benefit from biologics such as dupilumab, omalizumab, rituximab, and intravenous immunoglobulin (IVIg) therapy. A systematic review of case series comparing rituximab and anti-IgE therapy omalizumab saw comparable disease control with the two agents; however, those treated with rituximab had a lower relapse rate than those treated with omalizumab [9]. These therapies are acceptable with failed treatment response or if prednisone cannot be tapered below 10 mg per day with the use of secondary agents. While clinicians can trend BP180 and BP230 antibodies, the best signal of successful treatment is physical exam and status of lesions [5-8]. Notably, this patient had none of these recommended initial or step-up therapies prior to presentation, likely due to the lack of a clear working differential diagnosis. 

Pemphigus vulgaris treatment therapies differ from bullous pemphigoid treatment in that systemic glucocorticoids are the first-line treatment with tapering beginning once disease activity is clinically improved and remains improved with the lowest possible dose [1-13]. The acceptable criteria at which tapering can begin are when no new lesions have formed for at least seven days. Azathioprine adjuvant therapy has historically been used for steroid sparing and appears to have superior efficacy over mycophenolate for this purpose, though for the disease itself, azathioprine has an unclear efficacy on disease course [1-13]. No clinical randomized trials have studied the use of methotrexate in PV, and positive support for its use arises from uncontrolled retrospective reviews [1-13]. Refractory disease is often treated with lymphoma-dosed rituximab given the antibody-driven pathology [1,4-19]. There is also evidence suggesting that rituximab may be a beneficial initial treatment along with corticosteroids [1-8]. For faster disease control, rituximab has also been combined with IVIg or immunoadsorption therapy [1-9]. 

## Conclusions

The variable clinical presentation and complex overlap between bullous dermatoses poses a challenge for many clinicians and can result in delayed diagnoses or initiation of the proper treatment. Recognizing subtle cutaneous findings and prompt laboratory workup is essential to improve quality of life and decrease morbidity and mortality in these patients. Firstly, it is important to consider that BP can commonly present with mucosal lesions and presence of such should not preclude this diagnosis. Secondly, the presence of tense bullae among lesions is supportive of BP, even if bullae are in the minority of lesion findings. Similarly, the presence of widespread ulcerative and flaccid bullae as the majority of physical findings should not eliminate BP as a diagnosis, especially in the setting of intact bullae. These clinical considerations may provide more confidence in diagnosis and increase the time to optimal treatment initiation and improve patient outcomes.
